# *Amanitatullossiana*, a new species, and two new records of AmanitasectionLepidella from north-western Himalaya, India

**DOI:** 10.3897/mycokeys.37.26420

**Published:** 2018-07-31

**Authors:** Md. Iqbal Hosen, Tahir Mehmood, Kanad Das, Linas V. Kudzma, R. P. Bhatt

**Affiliations:** 1 State Key Laboratory of Applied Microbiology Southern China, Guangdong Provincial Key Laboratory of Microbial Culture Collection and Application, Guangdong Institute of Microbiology, Guangzhou 510070, China Guangdong Institute of Microbiology Guangzhou China; 2 Department of Botany and Microbiology, H.N.B. Garhwal University, Srinagar, Garhwal–246174, Uttarakhand, India H.N.B. Garhwal University Garhwal India; 3 Cryptogamic Unit, Botanical Survey of India, P.O. Botanic Garden, Howrah – 711103, India Cryptogamic Unit, Botanical Survey of India Howrah India; 4 37 Maple Avenue, Annandale, NJ 08801, USA Unaffiliated Annandale United States of America

**Keywords:** Amanitaceae, Basidiomycota, nrLSU, South Asian taxa, taxonomy

## Abstract

Amanitatullossiana, a new species of *Amanita* [subgenus Lepidella] section Lepidella from India is described. The species is characterised by its ash grey to brownish-grey pileus covered with dark grey to greyish-black universal veil remnants, the upper part of its rooting stipe base covered by several rows of recurved scales, broadly ellipsoid to ellipsoid basidiospores, absence of basidial clamp connections and pileal remnants of universal veil comprising abundant, disordered inflated cells intermixed with scattered filamentous hyphae. Molecular phylogenetic analysis and morphology both support the association of *A.tullossiana* with species of Bas’ stirps *Cinereoconia* – *A.cinereoconia* and *A.griseoverrucosa*. Two species, *A.griseoverrucosa* and *A.virgineoides* are reported here as new records for India.

*This article is dedicated to Dr. Rodham E. Tulloss for his contribution to mycology especially in the family Amanitaceae.*

## Introduction

The Amanitaceae is one of the most dominant and species-rich families of Basidiomycota. Traditionally, this family is divided into three genera, namely *Amanita* Pers., *Limacella* Earle and *Catatrama* Franco-Mol. However, a recent study by Redhead et al. (2016) divided *Amanita* into two genera, *Amanita* and *Saproamanita* Redhead, Vizzini, Drehmel & Contu, the former genus including species which are mycorrhizal in nature and the latter genus including only amycorrhizal/free-living species within *Amanita*. Subsequent to their establishment of the new genus, Tulloss et al. (2016) argued against the separation of *Saproamanita* from *Amanita* because the amycorrhizal species do not form a well-supported clade and are arguably the “mother” of the genus *Amanita* rather than a sister group within it. In this study, we follow the interpretation of Tulloss et al. (2016).

The Amanitaceae is characterised by longitudinally acrophysalidic stipe tissue. The agaricoid species in the genus *Amanita* are characterised by their schizohymenial development, which is evidence in mature basidiomata by their sterile lamella margin (Bas 1969, Thongbai et al. 2016, Tulloss et al. 2016, Bhatt et al. 2017).

The genus *Amanita* is divided into two subgenera: a) *Amanita* Pers. and b) *Lepidella* (E.-J. Gilbert) Veselý based on the reaction of basidiospore walls to Melzer’s reagent, the former having a negative reaction (inamyloid) and the latter having a positive reaction (amyloid) to that reagent (Corner and Bas 1962, Bas 1969, Yang 1997). The subg.Lepidella is further divided into four sections: i) sect. Amidella (J.-E Gilbert) Veselý, ii) sect. Lepidella sensu Bas (1969), iii) sect. Phalloideae (Fr.) Quél. and iv) sect. Validae (Fr.) Quél.

Species within Amanitasect.Lepidella are recognised by the combination of the following features: non-striate and appendiculate pileus margin and a volva that is friable, not forming an entire membranous sac (with the rare exception of a thin submembranous or membranous exterior layer). Approximately 200 taxa are listed for this section in the Amanitaceae website (http://www.amanitaceae.org/), of which 185 have been validly published (Corner and Bas 1962, Bas 1969, Tulloss and Jenkins 1985, Tulloss et al. 1992, Yang 1997, Wolfe et al. 2012, Deng et al. 2014, Cai et al. 2014, Li and Cai 2014, Hosen et al. 2015, Tulloss and Yang 2018). However, only four species, namely *A.albofloccosa* A.V. Sathe & S.D. Deshp., *A.berkeleyi* (Hooker f.) Bas, *A.eriophora* (Berk.) E.-J. Gilbert and *A.konkanensis* P.G. Sathe & S.M. Kulk. of Amanitasect.Lepidella have been reported from India so far (Bas 1969, Sathe and Daniel 1981, Kulkarni 1992).

During the course of macrofungal forays into different parts of the state of Uttarakhand, India, the second author (TM) collected several specimens of *Amanita* in broad-leaved forests. Morphological examination and molecular data indicated that the new collections herein reported represent one species new to science and two new records for India.

## Materials and methods

### Morphological study

Macromorphological characteristics were documented in the forest or base camp from fresh and dissected young to mature basidiomata. Photography was accomplished using a digital camera (Sony cyber-shot W730 and Cannon Power Shot SX 50). Colour codes follow Kornerup and Wanscher (1978). Samples were dried using an electric drier. Herbarium codes follow Index Herbariorum (Thiers 2018).

Micromorphological characteristics were observed with a compound microscope (Olympus CH20i) with dried material mounted in 5% KOH, 1% Phloxin, Melzer’s reagent and 1% Congo red. To present basidiospore measurements, the following notation was used: “[*n*/*m*/*p*]” indicating *n* basidiospores were measured from *m* basidiomata of *p* collections with a minimum of 20 basidiospores from each collection. Biometric variables followed those in Tulloss and Lindgren (2005): **L** = the range of the average spore length computed per specimen examined. **L**' = the average spore length computed for all spores measured. **W** = the range of the average spore width computed per specimen examined. **W**' = the average spore width computed for all spores measured. Q = the ratio of length/breadth for a single spore and the range of the ratio of length/breadth for all spores measured. **Q** = the average value of Q computed for one specimen examined and the range of such averages. **Q**' = average value of Q computed for all spores measured. *w_cs_* = the width of the central stratum of a lamella. *w_st_*-*near* = the distance from an outer margin of the central stratum to the nearest base of a basidium. *w_st_-far* = the distance from an outer margin of the central stratum to the furthest base of a basidium on the same side of the central stratum. Drawings of microscopic features were made free hand.

### Molecular study

DNA extraction, PCR amplification and sequencing

Genomic DNA was extracted from dry basidiomata following the modified CTAB method of Doyle and Doyle (1987). PCR was performed to amplify the partial sequence of the nuclear ribosomal large subunit (nrLSU) using universal primer pairs LR0R (GTACCCGCTGAACTTAAGC) and LR5 (ATCCTGAGGGAAACTTC) LR7 (TACTACCACCAAGATCT) (Vilgalys and Hester 1990) and the second largest subunit of RNA polymerase II (*rpb2*) using primer pair fRPB2-5F (GAYGAYMGWGATCAYTTYGG) (Liu et al. 1999) and bRPB2-7.1R (GCHATGGGKAARCARGCYATGGG) (Matheny 2005). Sequencing was performed on ABI 3730 XL DNA Analyzer (Applied Biosystems). PCR amplification (both nrLSU and *rpb2*) was conducted on a thermal cycler (Eppendorf, Germany) programmed for 3 min at 94 °C, followed by 35 cycles of 30 sec at 94 °C, 1 min at 55 °C, 1 min at 72 °C and a final stage of 8 min at 72 °C. The PCR products were purified using the QIAquick PCR Purification Kit (QIAGEN, Germany). Both strands of the PCR fragment were sequenced on the 3730*xl* DNA Analyzer (Applied Biosystems, USA) using the same primer pair.

### Phylogenetic analyses

In this study, a dataset of 49 nrLSU sequences of Amanitasubg.Lepidella and one nrLSU sequence of *Limacellabangladeshana* Iqbal Hosen were used for phylogenetic analysis. The nrLSU sequences of Amanitaceae were selected based on BLASTn search results (Altschul et al. 1997) and availability of sequences of Amanitaceae in GenBank (Clark et al. 2016). The nrLSU dataset was then aligned with Mafft v.6.8 (Katoh et al. 2005) and manually adjusted with BioEdit v.7.0.9 (Hall 1999) using default settings. Maximum Likelihood (ML) phylogenetic analysis inferred from nrLSU sequences was performed using RAxML v.7.2.6 (Stamatakis 2006). Default settings were used for all parameters in the ML analysis and statistical support values were obtained using nonparametric bootstrapping with 1,000 replicates. Gaps in the alignment were treated as missing data in the phylogenetic analysis. *Limacellabangladeshana* was selected as the outgroup for the molecular phylogenetic analysis.

## Results

### Molecular phylogenetic results

**Figure 1.**
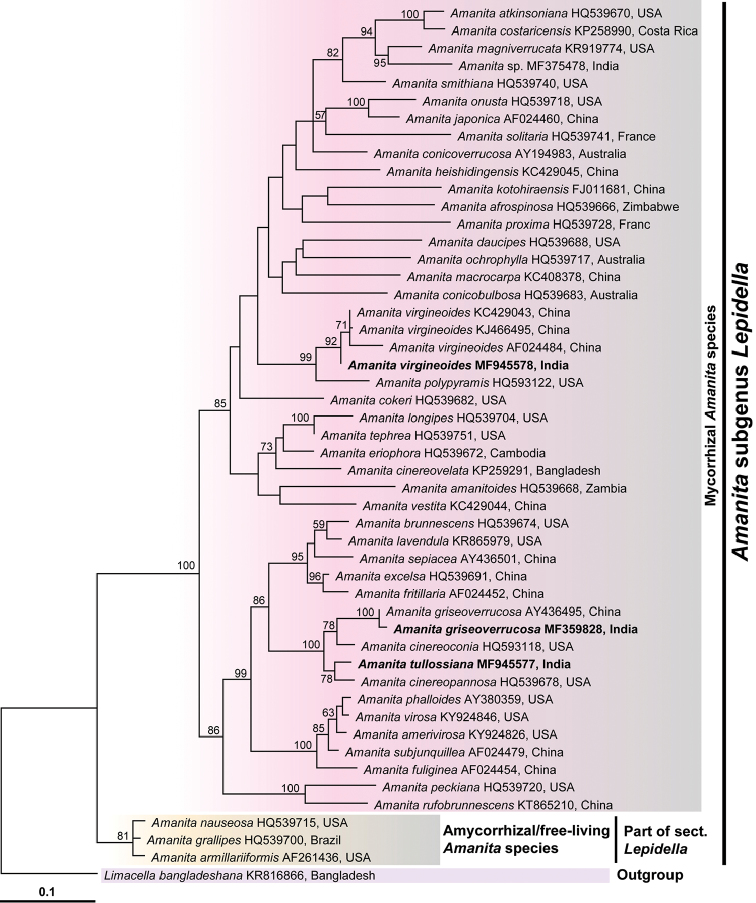
Phylogenetic relationships of *Amanitatullossiana*, *A.griseoverrucosa* and *A.virgineoides* inferred from nrLSU sequences using the Maximum Likelihood (ML) method. Bootstrap support values (≥50%) obtained from maximum likelihood (ML) analysis are shown above or beneath the branches at nodes. *Amanitatullossiana*, *A.virgineoides* and *A.griseoverrucosa* from India are highlighted in bold on the tree. GenBank accession numbers are provided after each species name and followed by country of origin.

In this study, five sequences (three for nrLSU and two for *rpb2*) were generated from three separate collections (RET 717-4, RET 717-9 and TM 16-1228) of *Amanita* and deposited in GenBank (Table 1). Only nrLSU sequences were used in this study to delimit the Indian *Amanita* species. The *rpb2* sequences were not used for reconstruction of molecular phylogeny because *rpb2* sequences for most of the *Amanita* species (included in the nrLSU phylogeny) are currently unavailable in GenBank for inclusion in this study. The aligned nrLSU dataset consisted of 50 sample sequences of Amanitaceae (Table 1) with 934 nucleotide sites for each sample (gaps included), of which 238 were parsimony informative characters. The resulting dataset was deposited in TreeBASE (S21668). Initial BLASTn search result of the nrLSU sequence of the Indian collection (RET 717-4) against the NCBI database exhibited 98% identity with *A.cinereopannosa* Bas (GenBank HQ539678) and 97% with *A.cinereoconia* G.F. Atk. (GenBank HQ593118). Phylogenetically, the collection RET 717-4 is grouped together with *A.cinereopannosa*, *A.cinereoconia* and *A.griseoverrucosa* Zhu L. Yang with strong bootstrap (BS) support (Fig. 1). Morphological characterisation [using the keys of Bas (1969)] and phylogenetic inference indicate the new collection (RET 717-4) is an independent species in *Amanita* [sect. Lepidellasubsect.Solitariae Bas] stirps *Cinereoconia* of Bas (1969). Another two collections TM 16-1228 and RET 717-9 are reported here as *A.griseoverrucosa* and A. virgineo*ides* Bas, respectively—new records to India. Phylogenetically, the former species is clustered with *A.cinereoconia*, *A.cinereopannosa* and *A.tullossiana* with strong support (100% ML BS); and the latter species is clustered with *A.polypyramis* (Berk. & M.A. Curtis) Sacc. (GenBank HQ593122, HQ539723) with strong support (99% ML BS) (Fig. 1).

**Table 1.** Taxa of Amanitaceae included in molecular phylogenetic analysis.

Name of the species	Herbarium voucher/collection/collector number	Geographic location	GenBank accession number	
			nrLSU	*rpb2*
* Amanita afrospinosa *	RET 347-1	Zimbabwe	HQ539666	–
* Amanita afrospinosa *	RET 347-1	Zimbabwe	HQ539666	–
* Amanita amanitoides *	RET 344-9	Zambia	HQ539668	–
* Amanita amerivirosa *	RET 628-2	USA	KY924826	–
*Amanita* sp.	TM 16-1247	India	MF375478	–
* Amanita armillariiformis *	DAOM216919	USA	AF261436	–
* Amanita atkinsoniana *	RET 301-1	USA	HQ539670	–
* Amanita brunnescens *	BW_HP12	USA	HQ539674	–
* Amanita cinereoconia *	BW_PSF	USA	HQ593118	–
* Amanita cinereopannosa *	RET 319-8	USA	HQ539678	–
* Amanita cinereovelata *	HKAS 81647^*^	Bangladesh	KP259291	–
* Amanita cokeri *	BW-STF 090506-19	USA	HQ539682	–
* Amanita conicoverrucosa *	–	–	AY194983	–
* Amanita costaricensis *	RET 330-4	Costa Rica	KP258990	–
* Amanita daucipes *	RET 386-8	USA	HQ539688	–
* Amanita eriophora *	RET 350-4	Cambodia	HQ539672	–
* Amanita excelsa *	Ge 816	China	HQ539691	–
* Amanita fritillaria *	HKAS 29511	China	AF024452	–
* Amanita fuliginea *	HKAS 32521	China	AF024454	–
* Amanita grallipes *	RET 379-5	Brazil	HQ539700	–
* Amanita griseoverrucosa *	HKAS 38459	China	AY436495	–
* Amanita griseoverrucosa *	TM 16-1228	India	**MF359828**	–
* Amanita heishidingensis *	HKAS 76122^*^	China	KC429045	–
* Amanita japonica *	HMAS 59778	China	AF024460	–
* Amanita kotohiraensis *	MHHNU 6998	China	FJ011681	–
* Amanita lavendula *	RET 339-7	Canada	KR865979	–
* Amanita longipes *	RET 360-1	USA	HQ539704	–
* Amanita magniverrucata *	RET 594-10	USA	KR919774	–
* Amanita macrocarpa *	31939L	China	KC408378	–
* Amanita nauseosa *	DPL 6117	USA	HQ539715	–
* Amanita ochrophylla *	PSC1127	Australia	HQ539715	–
* Amanita onusta *	RET 297-3	USA	HQ539718	–
* Amanita peckiana *	RET 320-3	USA	HQ539720	–
* Amanita phalloides *	Ben Woo (WTU)	USA	AY380359	–
* Amanita proxima *	RET 290-10	France	HQ539728	–
* Amanita polypyramis *	BW_CC	USA	HQ593122	–
* Amanita rufobrunnescens *	GDGM 42374^*^	China	KT865210	–
* Amanita sepiacea *	HKAS 38716	China	AY436501	–
* Amanita smithiana *	RET 382-6	USA	HQ539740	–
* Amanita solitaria *	RET 298-1	France	HQ539741	–
* Amanita subjunquillea *	HKAS 24169	China	AF024479	–
* Amanita tephrea *	RET 378-9	USA	HQ539751	–
* Amanita tullossiana *	RET 717-4^*^	India	**MF945577**	**MH638335^#^**
* Amanita vestita *	HKAS 77277	China	KC429044	–
* Amanita virgineoides *	RET 717-9	India	**MF945578**	**MH638336^#^**
* Amanita virgineoides *	HKAS 79691	China	KJ466495	–
* Amanita virgineoides *	HKAS 77278	China	KC429043	–
* Amanita virgineoides *	HKAS 18394	China	AF024484	–
* Amanita virosa *	RET 291-3	USA	KY924846	–
* Limacella bangladeshana *	Iqbal-276^*^	Bangladesh	KR816668	–

Newly generated sequences are highlighted in bold. ^*^Holotype. ^#^sequences (*rpb2*) derived from the new collections were not used in the phylogenetic tree but provided for future references. (–) indicates information is not available or not used in this study.

## Taxonomy

### Amanita tullossiana

Taxon classificationFungiAgaricalesAmanitaceae

Mehmood, Iqbal Hosen, K. Das & R.P. Bhatt, sp. nov.

822821

Figs 2 , 3

#### Typification.

INDIA, Uttarakhand, Rudhraparyag district, Baniyakund, at 2655 m a.s.l., 30°28.998N, 79°10.658E, 26 August 2014, T. Mehmood, TM 14-475 (RET 717-4, holotype; CAL 1611, isotype).

#### Etymology.

The epithet “*tullossiana*” (Lat., “of Tulloss”) is proposed in honour of Dr. Rodham E. Tulloss for his contribution to the study of the genus *Amanita* all over the world.

#### Diagnosis.

Distinct from all the known species of *Amanita* stirps *Cinereoconia* by the combination of the following characters: medium-sized to large basidiomata (pileus 90–170 mm wide, stipe 150–185 × 20–25 mm); brownish-grey to dark grey pileus covered with floccose to subfelted, pulverulent patches of universal veil remnants; broadly ellipsoid to ellipsoid basidiospores measuring (8.5–)9–13(–13.5) × (5.8–)6–8(–8.5) µm.

#### Description.

*Basidiomata* medium-sized to large. *Pileus* 90–170 mm wide, initially hemispherical then convex to plano-convex and finally planar, shiny, slightly viscid when moist, ash grey (1B2), pastel grey (1C1), grey (4B1-4C1), brownish-grey, brownish-beige (6F2-3) to dark grey (1F1), slightly darker at centre; context 11–14 mm thick above stipe, white (1A1), thinning evenly toward margin, unchanging when cut or bruised. *Universal veil on pileus* as floccose to subfelted pulverulent patches, dark grey (1F1) to brownish-grey (6F2), greyish-black to dark grey (1F1), soft, up to 4 mm thick, 7–12 mm wide, irregularly distributed. *Lamellae* 6–10 mm broad, free to narrowly adnate, crowded, white (1A1), unchanging when injured; lamellulae, plentiful of several lengths, attenuate, truncate, with 8–9 lamellae per cm at margin. *Stipe* 150–185 × 20–25 mm (excluding bulb), attenuate upwards, upper part covered by dark grey (1F1) fibrils, lower part covered with recurved scales, with fibrils turn blackish when handled; context solid, white, unchanging on cutting or bruising. *Partial veil* superior, soft, cottony, white, easily collapsed or detachable. *Bulb* 70–88 × 25–41 mm, napiform to rooting, covered with brownish-grey (6F2) to dark grey (1F1) universal veil remnants, often upper part covered with grey (4B1) to dark grey (1F1) recurving scales. *Odour* indistinct, *taste* not observed. *Spore deposit* white.

*Basidiospores* [300/15/10] (8.5–)9–13(–13.5) × (5.8–)6–8(–8.5) µm, [**L** = 9.5–11 µm, **L**' = 10.54 µm; **W** = 6–7.5 µm, **W**' = 6.83 µm; Q = (1.29–)1.40–1.66(–1.83), **Q** = 1.38–1.59, **Q**' = 1.54], broadly ellipsoid to ellipsoid, hyaline, thin-walled, smooth, amyloid; contents monoguttulate; apiculus lateral to sublateral, up to 1 µm long. *Basidia* 45–55(–65) × 9–14 µm, 2 to 4-spored, thin-walled; sterigmata up to 4 µm long; basal clamp connections absent. *Lamellar edge tissue* sterile, mainly composed of inflated globose to subglobose cells 20–35 × 15–25 µm and clavate to subclavate cells 40–50 × 15–18 µm. *Subhymenium* 40–50 μm thick, with 3–4 layers of inflated cells, *w_st_-near* = 35–50 μm, *w_st_-far*= 50–70 μm, basidia arising from small inflated cells 8–15 × 6–10 μm wide. *Hymenophoral trama* bilateral, divergent; *w_cs_*= 60–80 μm; well rehydrated, filamentous, undifferentiated hyphae 3–8 μm wide; with lateral stratum composed of intercalary inflated cells 66–110 × 12–19 μm wide; vascular hyphae 9–14 μm. *Pileipellis* 140–195 μm thick, in two layers, with gelatinised colourless suprapellis (45–55 μm) thick, filamentous, undifferentiated hyphae subradially arranged; subpellis (95–140 μm) thick; filamentous, undifferentiated hyphae 2–6 μm wide, densely arranged in subpellis, with yellowish-brown intracellular pigment; vascular hyphae 7–10 μm wide, infrequent. *Pileus context* filamentous, undifferentiated hyphae 2–6 μm wide, thin-walled, hyaline, interwoven; broadly clavate to ellipsoid cells 86–130 × 26–45 μm, thin-walled, hyaline. *Universal veil on pileus* disordered; filamentous, undifferentiated hyphae 2–6 μm wide, branched, thin-walled, infrequent to scattered, with pale yellow vacuolar pigments; inflated cells dominantly globose to subglobose 25–88 × 22–70 µm, infrequent broadly ellipsoid to ellipsoid or pyriform 40–60 × 10–13 μm, often in chains of 2–3, with brownish to pale yellow vacuolar pigments; vascular hyphae 6–12 μm wide, frequent. *Universal veil on stipe base* disordered; filamentous, undifferentiated hyphae 2–5 μm wide, branched, thin-walled, scattered, with pale yellow vacuolar pigments; inflated cells dominantly globose to subglobose 30–70 × 25–65 µm, infrequent broadly ellipsoid to elongated cells 30–90 × 12–18 μm, with brownish to pale yellow vacuolar pigments; vascular hyphae 10–14 μm wide, often present. *Partial veil* abundant inflated cells broadly clavate to clavate 50–120 × 16– 29 µm, thin-walled, colourless, hyaline, sometimes with yellowish-brown vacuolar pigments; filamentous, undifferentiated hyphae 3–7 µm wide, dominant, thin walled, hyaline, colourless or sometimes with yellowish-brown pigments; vascular hyphae 4–8 μm wide. *Stipe context* longitudinally acrophysalidic; filamentous, undifferentiated hyphae 5–7 µm wide; acrophysalides 150–230 × 35–56 µm, thin-walled, colourless, hyaline, vascular hyphae not found. *Clamp connections* not observed in any tissues.

**Figure 2.**
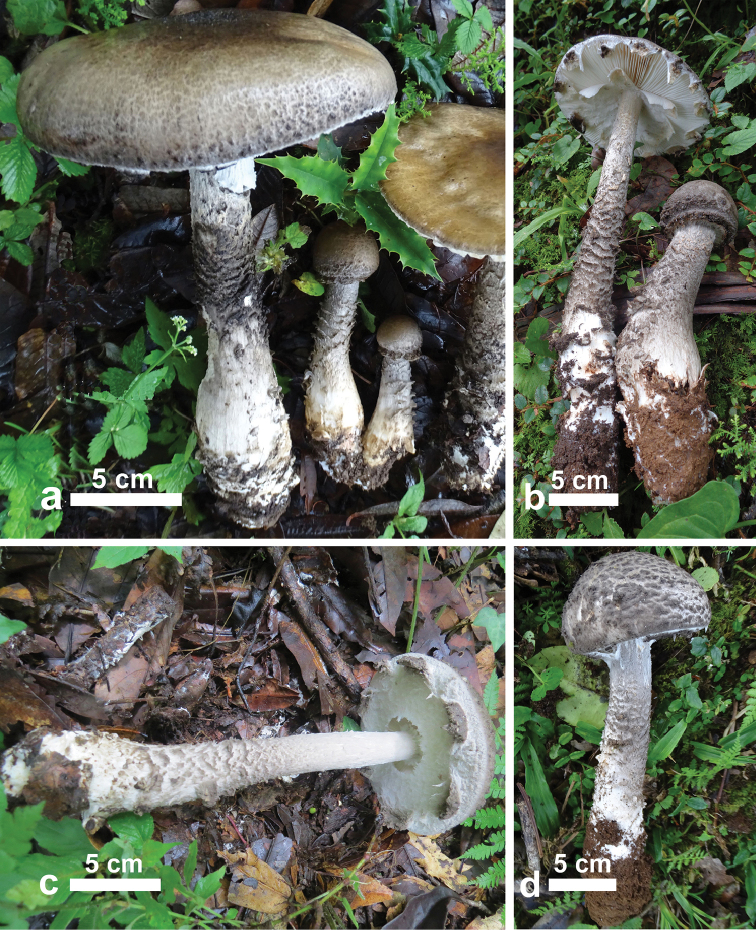
Basidiomata of *Amanitatullossiana* in natural habitat (RET 717-4, holotype; CAL 1611, isotype). **a–d** showing distinctive features of *A.tullossiana* (universal veil remnants, appendiculate pileus margin and recurved scales on the stipe surface).

**Figure 3.**
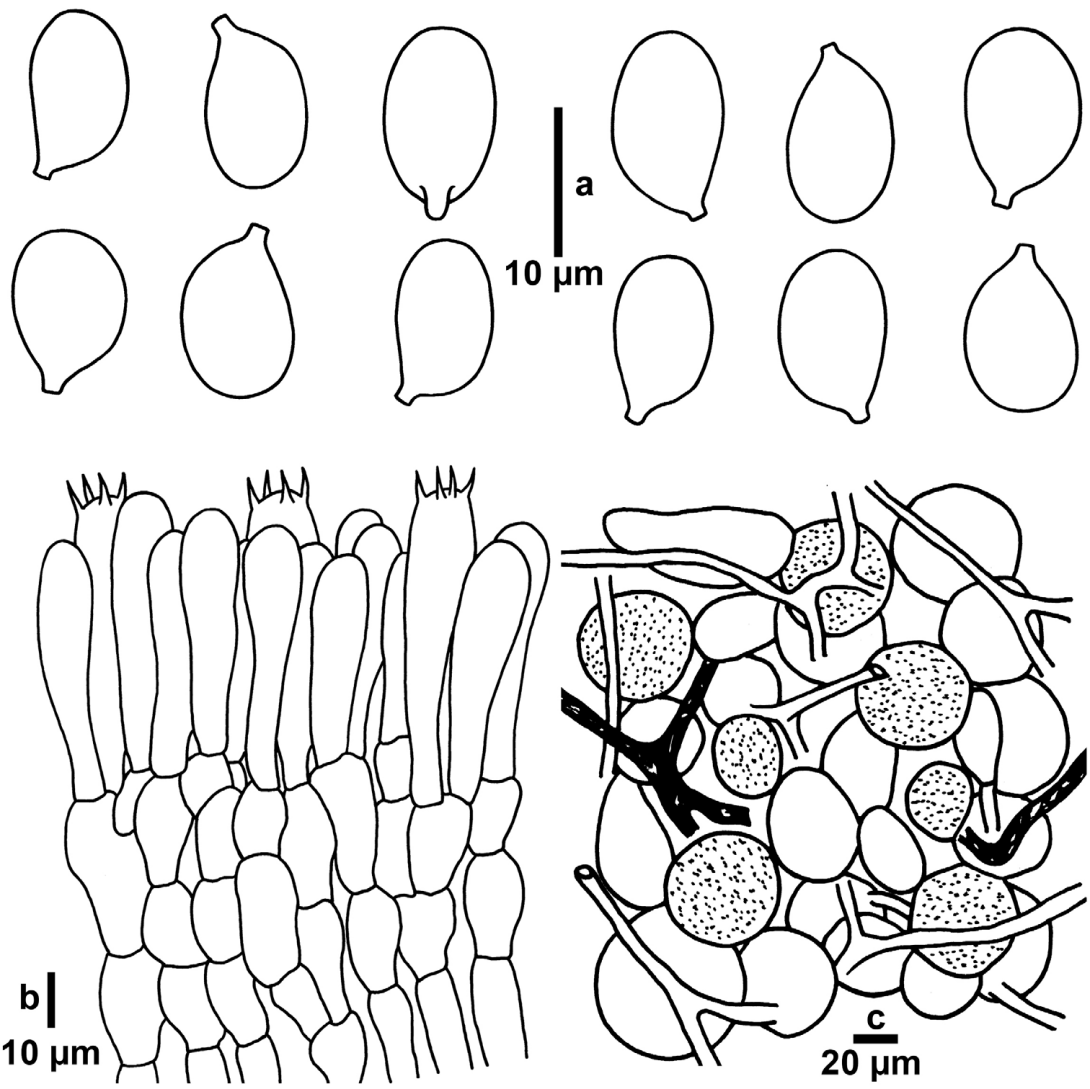
Microscopic features of *Amanitatullossiana* (RET 717-4, holotype; CAL 1611, isotype). **a** Basidiospores **b** Basidia at different stages of development **c** Elements of universal veil from pileus surface (vascular hyphae are dark shaded).

#### Macrochemical tests on fresh basidiomata.

5% KOH - negative on pileus, 2% phenol - negative and FeSO4 crystals - negative on pileus and in stipe context.

#### Habitat and distribution.

Solitary to subgregarious in temperate mixed forest dominated by *Quercussemicarpifolia* and *Abiespindrow*, at 2350–2655 m a.s.l. Currently only known from India.

#### Additional specimens examined.

INDIA, Uttarakhand, Rudraparyag district, Baniyakund, 26 August 2014, T. Mehmood, TM 14-486 (GUH-M-27001); same location, 14 July 2015, T. Mehmood, TM 15-624 (GUH-M-27002); same location, 1 August 2015, T. Mehmood, TM 15-786 (GUH-M-27003); same location, 2 August 2015, T. Mehmood, TM 15-815 (GUH-M-27004); same location, 8 August 2015, T. Mehmood, TM 15-891 (GUH-M-27005); same location, 30 August 2015, T. Mehmood, TM 15-1017 (GUH-M-27006); same location, 22 July 2016, T. Mehmood, TM 16-1123 (GUH-M-27007); same location, 26 August 2016, T. Mehmood, TM 16-1369 (GUH-M-27008); Nainital district, Mukteshwar 24 August 2016, T. Mehmood, TM 16-1338 (GUH-M-27009).

#### Commentary.

The grey to brownish-grey universal veil, the absence of clamp connections, disordered inflated cells intermixed with scattered filamentous hyphae, together with broadly ellipsoid to cylindrical basidiospores are the key features of sect. Lepidella stirps *Cinereoconia* (Bas 1969). Based on the Bas’ key, the new taxon could be placed in *Amanita* [sect. Lepidellasubsect.Solitariae] stirps *Cinereoconia*.

In stirps *Cinereoconia*, *A.griseofarinosa* Hongo, *A.lutescens* Hongo, *A.pelioma* Bas, *A.odorata* Beeli, *A.vestita* Corner & Bas, *A.griseovelata* D.A. Reid, *A.pallidoflavescens* Dav. T. Jenkins and A. viri*dissima* Wartchow are all species that should be compared to the morphology of the present taxon. *Amanitagriseofarinosa*, originally described from Japan, has a pale yellowish-grey pileus covered with dark coloured, farinose to tomentose universal veil remnants; and subglobose to broadly ellipsoid basidiospores 8.5–10 × 7–9 μm, with a lower **Q**' value = 1.2 (Bas 1969) than the basidiospores of the present taxon. *Amanitalutescens*, originally described from Japan, differs from *A.tullossiana* by its small to medium-sized basidiomata 35–60 mm broad, context turning yellowish when cut or bruised and relatively smaller basidiospores 8–10(–10.5) × 5.5–6.5 μm (Bas 1969). *Amanitapelioma*, originally described from the USA, has a greyish-olive to pale brownish pileus, distinctive brown gills, a volva that bruises a distinctive blue-green and ellipsoid to elongate basidiospores 10–12.5 × 6.5–8 µm, with a higher **Q**' value = 1.65 (Bas 1969) than in the new species. *Amanitaodorata*, originally described from the Democratic Republic of Congo, has a greyish olivaceous brown pileus, pinkish-white lamellae and elongate to cylindric basidiospores 9.5–13 × 4.5–5.5 µm, with a **Q** value ranges = 1.55–2.05 (Bas 1969). *Amanitavestita*, originally described from Singapore, has a pale greyish-white pileus covered with small micaceous umber particles, broadly ellipsoid to ellipsoid basidiospores 7.5–9 × 5.5–6.5 µm, with a **Q** value ranges = 1.3–1.35 (Bas 1969) lower than in the new taxon. *Amanitagriseovelata*, originally described from Victoria, Australia, has a slate-grey pileus covered pale grey, felty-pruinose universal veil remnants and subglobose to broadly ellipsoid basidiospores 7–10(–11.5) × 6.8–8.5 μm (Reid 1980). *Amanitapallidoflavescens*, originally described from the USA, has a white to silvery white pileus and bears elongate to cylindric basidiospores 8.6–10.2 × 4.7–5.5 µm (Jenkins 1980). *Amanitaviridissima*, originally described from Brazil, has a green pileus and stipe, pale lamellae and elongate to cylindric basidiospores 9.8–13 × 5.7–8.3 µm, with a higher **Q**' value =1.82 (Wartchow 2016).

*Amanitacinereopannosa*, *A.cinereoconia* and *A.griseoverrucosa* are the phylogenetically closely related species to the new species (Fig. 1). However, all of them are distinguished morphologically. *Amanitacinereopannosa*, originally described from USA, has a white to silvery sheen pileus covered with subfelted to subpyramidal warts, abundant filamentous hyphae and ellipsoid to elongated basidiospores (8–)8.8–10(–14.1) × (4.9–)5–6.7(–8.3) µm (Tulloss and Yang 2018). Furthermore, this species is considered endemic to eastern North America and has not been recorded in other parts of the world (Davison et al. 2013). Bas (1969) clearly held *A.cinereopannosa* to be distinct from the species of stirps *Cinereoconia* because he placed it in his stirps *Strobiliformis*. *Amanitacinereoconia*, originally described from the USA, has a white to greyish pileus covered with grey, pulverulent to small warted universal veil remnants and bears elongate to cylindric basidiospores 7.8–10.9 × 4.7–6.2 µm, with a **Q** value = 1.72 (Jenkins 1986). In addition, *A.cinereoconia* has a peculiar smell like “chloride of lime” [meaning the smell of an outdoor pit toilet into which CaCl_2_ has been added; hence, an odour of decaying protein] or faintly of “chlorine” (Bas 1969; Jenkins 1986). Bas proposed a variety *croceescens* of *A.cinereoconia*; however, Tulloss had the opportunity to observe the transition of a single specimen from the “type variety” to “var. croceescens” and attributed the yellow colouration to the *Amanita* “yellowing syndrome” (Tulloss, pers. comm.). *Amanitagriseoverrucosa*, originally described from China and reported here from India (see below), has a dirty white to greyish pileus, verrucose to conical universal remnants, a white to greyish-white stipe, a ventricose to clavate bulb and relatively smaller spores measuring 8–11 × 5.5–7 μm (Yang 2004) in comparison to *A.tullossiana* 9–13 × 6–8 μm.

### Amanita griseoverrucosa

Taxon classificationFungiAgaricalesAmanitaceae

Zhu L. Yang, Bibliotheca Mycologica 170: 155 (1997)

Figs 4a, b , 5a, b

#### Description.

*Basidiomata* medium-sized to large. *Pileus* 60–125 mm wide, initially hemispherical then convex to plano-convex, dry, slightly viscid when moist, whitish to greyish-white (1B1) to ash grey (1B2) to grey (1D1); context 6–11 mm thick, white (1A1), thinning evenly towards margin, unchanging when cut or bruised. *Universal veil on pileus* as felted to subconical to verrucose, brownish-grey (1D3), greyish-brown (5F3) to dark grey (1F1), soft, up to 4 mm thick, 5–8 mm wide, irregularly distributed; margin non-striate, appendiculate; *Lamellae* free to narrowly adnate, crowded, white (1A1), unchanging, 6–10 mm broad; lamellulae attenuate, plentiful, of several lengths, with 7–8 lamellae per cm at margin. *Stipe* 45–90 × 12–21 mm (excluding bulb), narrowing upwards, solid, lower part covered by light grey (1D1) fibrillose squamules, upper part covered by white farinose squamules; context white, unchanging on cutting or bruising. *Bulb* 32–62 × 19–32 mm, ventricose to clavate, white, covered with grey (1D1) to dark grey (1F1), universal veil remnants. *Partial veil* superior, soft, cottony, white, easily collapsed. *Odour* indistinct, *taste* not observed. *Spore deposit* white.

*Basidiospores* [80/4/2] (8–) 8.5–10(–11) × (5.5–)6 –6.5 (–7) µm, [**L** =9.05–9.17 µm, **L**' = 9.11 µm; **W** = 5.9–6.5 µm, **W**' = 6.2 µm; Q = (1.32–)1.42–1.5(–1.69), **Q** = 1.51–1.54, **Q**' = 1.53], ellipsoid, hyaline, thin walled, smooth, amyloid, apiculus sublateral, up to 1 µm. *Basidia* (34–)45–50(–53) × (9.5–)10–12(–14) µm, 2 to 4-spored, thin-walled, colourless, hyaline; sterigmata up to 4 µm long; basal clamp connections not observed in any tissue after extensive search. *Lamellae edge* sterile; composed of clavate or pyriform inflated cells 35–50 × 22–31 μm, thin walled, colourless, hyaline. *Subhymenium* 35–40 μm thick, *w_st_-near* = 30–40 μm, *w_st_-far* = 40–55 μm, basidia arising from subglobose to broadly ellipsoid cells (11–18 × 8–15 μm). *Hymenophoral trama* bilateral, divergent; *w_cs_* = 40–60 μm; well rehydrated, filamentous, undifferentiated hyphae 3–8 μm wide; inflated cells ellipsoid to elongated 55–90 × 12–19 μm, diverging at an angle of approximately 40°; vascular hyphae 11–14 μm wide, infrequent. *Pileipellis* 130–150 μm thick, subradially to densely arranged, filamentous, undifferentiated hyphae 2–7 μm wide; vascular hyphae 7–10 μm wide, infrequent. *Universal veil on pileus* disordered; filamentous, undifferentiated hyphae 2–7 μm wide, scattered, branched, thin walled; inflated cells dominantly globose to subglobose 40–70 × 30–65 µm, broadly ellipsoid to ellipsoid 40–60 × 10–13 μm, often in chain of 2–3 cells, thin walled, hyaline, often with yellowish-brown vascular pigment. *Universal veil on base of stipe* disordered; filamentous, undifferentiated hyphae 3–8 μm wide, scattered, thin walled, branched, with brownish vacuolar pigments; inflated cells dominantly globose to subglobose 30–65 × 26–58 µm, broadly ellipsoid to ellipsoid or pyriform 26–55 × 8–13 μm, thin-walled, hyaline, with brownish vacuolar pigment. *Partial veil* abundant inflated cells clavate to broadly clavate 76–130 × 13–25 µm, thin walled, colourless, hyaline or brownish vacuolar pigments; filamentous, undifferentiated hyphae 3–5 µm wide. *Stipe context* longitudinally acrophysalidic, filamentous, undifferentiated hyphae 5–7 µm wide; acrophysalides 220–270 × 33–45 µm, filamentous, undifferentiated hyphae 4–8 µm wide, hyaline, vascular hyphae not found. *Clamp connections* not observed in any tissue.

**Figure 4.**
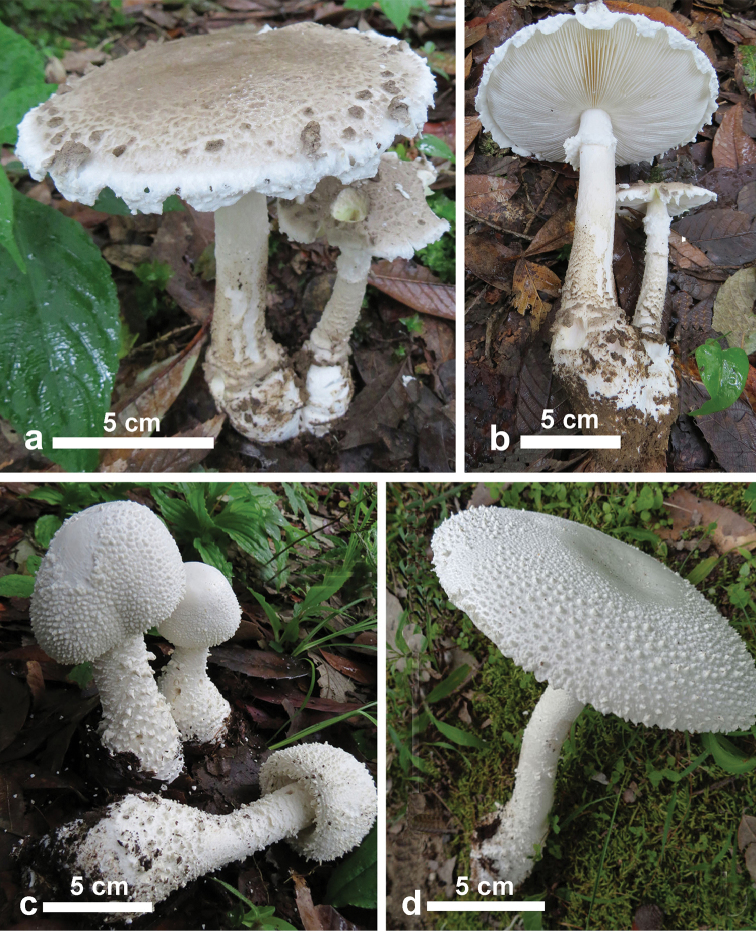
Basidiomata of *Amanita* species. **a, b** Basidiomata of *A.griseoverrucosa* in natural habitat (TM 16-1228) **c, b** Basidiomata of *A.virgineoides* in natural habitat (TM 14-413).

**Figure 5.**
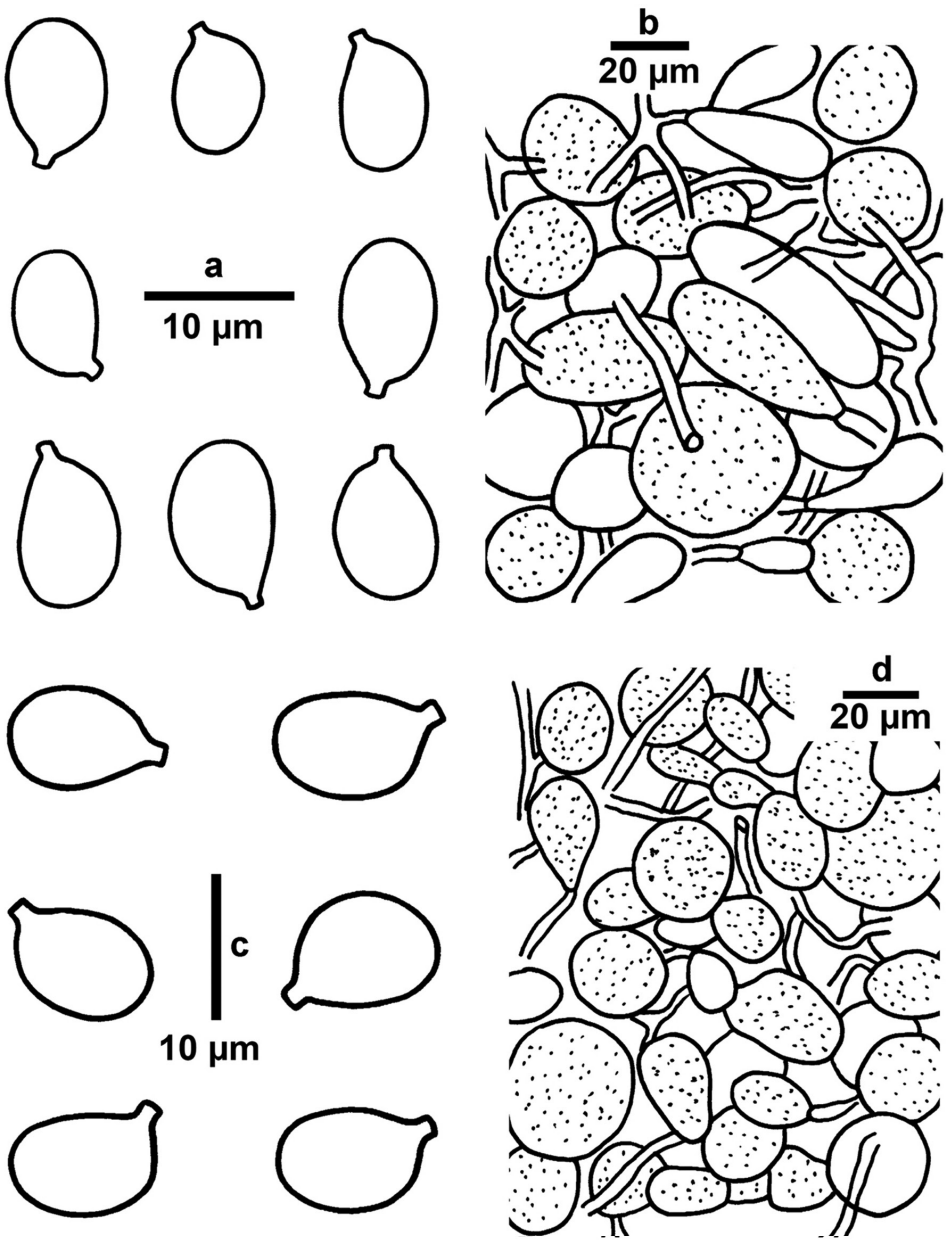
Microscopic features of *Amanita* species. **a, b***Amanitagriseoverrucosa* (TM 16-1247) **a** Basidiospores **b** Elements of universal veil from pileus surface **c, d***Amanitavirgineoides* (TM 14-413) **c** Basidiospores **d** Elements of universal veil from pileus surface.

#### Habitat and distribution.

Solitary to gregarious, with plants of Fagaceae, Pinaceae and Ericaceae (*Rhododendronarboretum*).

#### Known distribution.

Currently known from China (Yang 2004, 2015) and now India.

#### Specimens examined.

INDIA, Uttarakhand, Pauri district, Phedkhal, at 1900 m a.s.l., 30°09.728'N, 078°51.206'E, 29 July 2016, T. Mehmood, TM 16-1228 (GUH-M-27010); same location, 26 August 2015, T. Mehmood, TM-15-971 (GUH-M-27011), 1910 m a.s.l., 30°09.732'N, 078°51.214'E.

#### Commentary.

Morphologically, the Indian collections of *A.griseoverrucosa* are characterised by a whitish to greyish-white pileus covered with easily detachable greyish-brown to dark grey, felted to verrucose universal veil remnants, a ventricose to clavate stipe base, broadly ellipsoid to ellipsoid basidiospores, universal veil on the pileus with abundant inflated cells and scattered filamentous, undifferentiated hyphae and the absence of clamp connections at bases of basidia. The characteristic features and molecular data from the Indian collections match rather well with the original description of *A.griseoverrucosa*, reported from China (Yang 2004).

The absence of clamp connections at the bases of basidia, ellipsoid to broadly ellipsoid basidiospores and abundant inflated cells with scattered hyphae in the universal veil placed this species in *Amanita* [sect. Lepidellasubsect.Solitariae] stirps *Cinereoconia* (Yang 2004). Phylogenetically, both Indian (TM 16-1228) and Chinese (HKAS 38459) collections of *A.griseoverrucosa* are closely related to *A.cinereoconia* and *A.tullossiana* (Fig. 1). *Amanitacinereoconia* has a white to greyish pileus covered with pulverulent to small warted universal veil remnants and elongate to cylindric basidiospores 8.5–11.5 × 5–6.5 µm (Bas 1969, Jenkins 1986). *Amanitagriseoverrucosa* is also distinguished from *Amanitatullossiana* (see above).

### Amanita virgineoides

Taxon classificationFungiAgaricalesAmanitaceae

Bas, Persoonia 5: 435 (1969)

Figs 4c, d , 5c, d

#### Description.

*Basidiomata* medium-sized to large. *Pileus* 50–140 mm wide, white to slightly yellowish-white (1A2) with age, ovoid at first, hemispherical when expanding, later convex to plano-convex to flat; slightly depressed, dry, shiny, densely covered with conical to subconcal warts; margin appendiculate, incurved; context 8–13 mm thick, thinning evenly towards margin, white, turning yellowish-white (1A2) when cut or bruised. *Universal veil on pileus* as conical, subconic to pyramidal warts, 5–10 mm thick, white, easily detachable when touched, sometimes washed away by rains, turning slightly yellowish-white (1A2) with age. *Lamellae* 12–15 mm thick, free, white (17A1) crowded, with 8–9 lamellae per cm at margin; lamellulae attenuate, of 4–5 lengths, plentiful, white to cream. *Stipe* 75–140 × 26–22 mm (excluding bulb), white (16A1), slightly tapering upwards, the upper part covered by flocculent squamules, the lower part covered by irregularly arranged, conical to sub-conical warts; context white, solid, turning light yellowish (1A3) when cut or bruised. *Bulb* 23–29 × 23–30 mm, subglobose, ovoid to napiform, white, slightly yellowish-white with age. *Universal veil on stipe base* as white conical to subconical warts. *Partial veil* superior, white, submembranous, thick, covered with white conical warts, fragile, easily detachable when touched. *Odour* unpleasant. *Taste* not recorded. *Spore print* white.

*Basidiospores* [180/9/4] (7.5–)8–10.5(–11) × (5.5–)5.8–7.5 µm, [**L** = 8–10 µm, **L**' = 9.05 µm; **W** = 6.0–6.7 µm, **W**' = 6.45 µm; Q = (1.22–)1.33–1.55(–1.66), **Q** = 1.33–1.46, **Q**' = 1.41], colourless, hyaline, thin walled, smooth, amyloid, broadly ellipsoid to ellipsoid; apiculus lateral to sublateral, up to 1 µm long; contents monoguttulate. *Basidia* (42–)48–51(–58) × (10–)11–12(–12.5) µm, 2 to 4-spored, thin-walled, colourless, hyaline; sterigmata up to 4 µm long; basal septa often clamped. *Lamellar edge* tissue sterile, with inflated cells; subglobose to pyriform 15–25 × 8–15) μm, thin walled, colourless, hyaline, clamps present. *Subhymenium* 30 µm thick, *w_st_-near* = 28–45 μm, *w_st_-far*= 35–50 μm, ramose, with inflated; ovoid to ellipsoid cells 12–18 × 8–14 μm; clamp present. *Hymenophoral trama*, bilateral, divergent; *w_cs_*= 40–65 μm; lateral stratum comprising of inflated intercalary segment 30–65 × 8–20 μm, common; filamentous, undifferentiated hyphae 3–9 μm wide, thin-walled, colourless, hyaline, vascular hyphae rare; clamp present. *Pileipellis* hardly differentiated; filamentous hyphae 2–7 μm wide, interwoven, non-gelatinised, thin walled, colourless, hyaline. *Universal veil on the pileus* with elements anticlinally arranged; filamentous, undifferentiated hyphae 4–8 μm wide, abundant, branched, colourless, hyaline; inflated cells dominantly subglobose to pyriform 16–46 × 14–32 μm, broadly ellipsoid to fusiform 30–66 × 10–21 μm; clamp present. *Universal veil on the stipe base* with elements anticlinally arranged; filamentous, undifferentiated hyphae 4–7 µm wide, scattered to abundant, colourless, thin walled, hyaline; inflated cells dominantly globose to subglobose 20–50 × 18– 48 µm, broadly ellipsoid to ellipsoid 45–65 × 15–20 µm, thin walled, hyaline, colourless, clamps present. *Partial veil* abundant inflated cells subglobose to ellipsoid 15–36 × 12–28 μm, thin walled, colourless, hyaline; filamentous, undifferentiated hyphae 3–8 µm wide, dominant, colourless, thin walled, clamps present. *Stipe context* longitudinally acrophysalidic; filamentous hyphae 2–13 μm wide, acrophysalides measuring 120 –181 × 20–30 μm, dominant, colourless, thin walled, hyaline, clamps present. *Clamp connections* common.

#### Macrochemical tests on fresh basidiomata.

Chemical reactions on pileus surface: 10% NH_4_OH - pinkish, 5% KOH - negative, 2% phenol - negative; and FeSO_4_ crystals - negative on pileus and stipe context.

#### Habitat and distribution.

Solitary to subgregarious in temperate mixed forest dominated by *Quercus leucotrichophora and Cedrus deodara* at 1850–2050 m a.s.l.

*Known distribution*: This species was originally described from Japan. It has also been reported from China (Yang 1997), South Korea (Kim et al. 1993), Thailand (Sanmee et al. 2008) and now India.

#### Specimens examined.

INDIA, Uttarakhand, Pauri district, Phedkhal, 24 August 2014, T. Mehmood, TM 14-413 (RET 717-9); same location, 12 August 2015, T. Mehmood, TM 15-917 (GUH-M-27012); same location, 16 July 2016, T. Mehmood, TM 16-1098 (GUH-M-27013); same location, 24 July 2017, T. Mehmood, TM 17-1468 (GUH-M-27014).

#### Commentary.

An Indian collection (RET 717-9) is grouped phylogenetically with Chinese material of *A.virgineoides* (HKAS 79691, GenBank nrLSU: KJ466495 and HKAS 77278, GenBank nrLSU: KC429043), with pairwise genetic divergence between their nrLSU sequences = 0.35% (might be intragenomic heterogeneity present amongst collections as the sequence was not clean). It is worth mentioning that there is no genetic distance between *rpb2* sequences derived from the Chinese (HKAS 79691, GenBank *rpb2*: KJ466663) and Indian (RET 717-9) collections. The evidence suggests that the two collections could be conspecific and exhibiting a minor intra-specific variability. In addition, the sample size is also small. For these reasons, we do not feel justified in erecting a new species or subspecies. Interestingly, another Chinese collection (HKAS 18394), labelled as *A.virgineoides* (GenBank nrLSU: AF024484, Weiß et al. 1998), is also grouped with the Indian collection, but the sequence derived from this collection is divergent from the two previously cited collections (Fig. 1). However, the habit and size of the basidiomata and basidiospores of the Indian collections match well with those characters in the descriptions of *A.virgineoides* provided by Bas (1969) and Yang (1997, 2015). Therefore, the Indian collection (RET 717-9) is being treated here as *A.virgineoides* – a new record for India.

*Amanitavirgineoides* belongs to *Amanita* [sect. Lepidellasubsect.Solitariae] stirps *Virgineoides* because of the presence of conical to subconical warts on the pileus surface which consist of inflated cells rather abundant hyphae, the presence of clamp connections at the bases of basidia and the broadly ellipsoid basidiospores (Bas 1969, Yang 1997). In stirps *Virgineoides*, *A.gracilior* Bas & Honrubia and *A.miculifera* Bas & Hatanaka resemble *A.virgineoides* morphologically. *Amanitagracilior*, originally described from Spain, has a white pileus turning yellowish-brown with age, a rooting base and elongate basidiospores 10–11.5 × 5.5–6.5 µm, with a higher **Q**' value = 1.8 (Bas 1969). *Amanitamiculifera*, originally described from Japan, has a pearl grey pileus and a stipe with a notably radicating basal bulb (Bas and Hatanaka 1984, Yang 1997). The white basidiomata of *A.virgineoides* also resembles the basidiomata of other of Bas’ stirpes. In creating these stirpes, Bas morphologically segregated these taxa from *A.virgineoides* (Bas 1969).

## Supplementary Material

XML Treatment for Amanita tullossiana

XML Treatment for Amanita griseoverrucosa

XML Treatment for Amanita virgineoides

## References

[B1] Altschul SF Madden TL Schaffer AA Zhang J Zhang Z Miller W Lipman DJ (1997) Gapped BLAST and PSI-BLAST: a new generation of protein database search programs. Nucleic Acids Research 25: 3389–3402. 10.1093/nar/25.17.3389 9254694 PMC146917

[B2] Bas C (1969) Morphology and subdivision of *Amanita* and monograph of its section Lepidella. Persoonia 5: 285–579.

[B3] Bas C Hatanaka SI (1984) An undescribed species of AmanitasectionLepidella from Japan. Persoonia 12: 321–325.

[B4] Bhatt RP Mehmood T Uniyal P Singh U (2017) Six new records of *Amanita* (Amanitaceae) from Uttarakhand, India. Current Research in Environmental & Applied Mycology 7: 161–182. 10.5943/cream/7/3/3

[B5] Cai Q Tulloss RE Tang LP Tolgor B Zhang P Chen ZH Yang ZL (2014) Multi-locus phylogeny of lethal amanitas: implications for species diversity and historical biogeography. BMC Evolutionary Biology 14: 1–31. 10.1186/1471-2148-14-143 24950598 PMC4094918

[B6] Clark K Karsch-Mizrachi I Lipman DJ Ostell J Sayers EW (2016) GenBank. Nucleic Acids Research 44: D67–D72. 10.1093/nar/gkv1276 PMC4702903 26590407

[B7] Corner EJH Bas C (1962) The genus *Amanita* in Singapore and Malaya. Persoonia 2: 241–304.

[B8] Davison EM McGurk LE Bougher NL Syme K Watkin EL (2013) *Amanitalesueurii* and *A.wadjukiorum* (Basidiomycota), two new species from Western Australia, and an expanded description of *A.fibrillopes*. Nuytsia 23: 589–606.

[B9] Deng WQ Li TH Li P Yang ZL (2014) A new species of AmanitasectionLepidella from South China. Mycological Progress 13: 211–217. 10.1007/s11557-013-0906-6

[B10] Doyle JJ Doyle JL (1987) A rapid DNA isolation procedure for small quantities of fresh leaf tissue. Phytochemical Bulletin 19: 11–15.

[B11] Hall TA (1999) BioEdit: a user-friendly biological sequence alignment editor and analysis program for Windows 95/98/NT. Nucleic Acids Symposium Series 41: 95–98.

[B12] Hosen MI Li TH Deng WQ (2015) *Amanitacinereovelata*, a new species of AmanitasectionLepidella from Bangladesh. Mycological Progress 14: 35. 10.1007/s11557-015-1058-7

[B13] Jenkins DT (1986) *Amanita* of North America. Mad River Press, Eureka. California, 198 pp.

[B14] Katoh K Kuma K Toh H Miyata T (2005) MAFFT version 5: improvement in accuracy of multiple sequence alignment. Nucleic Acids Research 33: 511–518. 10.1093/nar/gki198 15661851 PMC548345

[B15] Kim YS Suck SJ Park YH Cha DY (1993) *Amanita* in Korea. Proceedings of the First Korea-China Joint Seminar for Mycology. Seoul, Dec. 2–5, 114–127.

[B16] Kornerup A Wanscher JH (1978) Methuen handbook of colour. 3rd edn. Methuen, London.

[B17] Kulkarni SM (1992) *Amanitakonkanensis*: a new species of Agaricales. Biovigyanam 18: 56–58.

[B18] Li F Cai Q (2014) *Amanitaheishidingensis*, a new species of Amanitasect.Lepidella from China. Mycological Progress 13: 1191–1197. 10.1007/s11557-014-1008-9

[B19] Liu YL Whelen S Hall BD (1999) Phylogenetic relationships among ascomycetes: evidence from an RNA polymerase II subunit. Molecular Biology and Evolution 16: 1799–1808. 10.1093/oxfordjournals.molbev.a026092 10605121

[B20] Matheny PB (2005) Improving phylogenetic inference of mushrooms with *RPB1* and *RPB2* nucleotide sequences (*Inocybe*; Agaricales). Molecular Phylogenetics and Evolution 35: 1–20. 10.1016/j.ympev.2004.11.014 15737578

[B21] Redhead SA Vizzini A Drehmel DC Contu M (2016) *Saproamanita*, a new name for both *Lepidella* E.-J. Gilbert and *Aspidella* E.-J. Gilbert (Amaniteae, Amanitaceae). IMA fungus, 7: 119–129. https://doi: 10.5598/imafungus.2016.07.01.07 27433443 PMC4941681

[B22] Reid DA (1980) A monograph of the Australian species of *Amanita* Pers. ex Hook. (Fungi). Australian Journal of Botany Supplementary Series 8: 1–97.

[B23] Sanmee R Tulloss RE Lumyong P Dell B Lumyong S (2008) Studies on *Amanita* (Basidiomycetes: Amanitaceae) in Northern Thailand. Fungal Diversity 32: 97–123.

[B24] Sathe AV Daniel J (1981) Agaricales (Mushrooms) of Kerala State in Agaricales of South West India. Maharashtra Assoc. for the Cultivation of Science, Pune, 75–108.

[B25] Stamatakis A (2006) RAxML-VI-HPC: maximum likelihood-based phylogenetic analyses with thousands of taxa and mixed models. Bioinformatics 22: 2688–2690. 10.1093/bioinformatics/btl446 16928733

[B26] Thiers BM (2018) (mutable text) Index Herbariorum. http://sciweb.nybg.org/science2/IndexHerbariorum.asp [Accessed 12 July 2018]

[B27] Thongbai B Tulloss RE Miller SL Hyde KD Chen J Zhao R Raspé O (2016) A new species and four new records of *Amanita* (Amanitaceae; Basidiomycota) from Northern Thailand. Phytotaxa 286: 211–231. 10.11646/phytotaxa.286.4.1

[B28] Tulloss RE Jenkins DT (1985) Validation of *Amanitalongipes*. Mycotaxon 22: 439–442.

[B29] Tulloss RE Kuyper TW Vellinga EC Yang ZL Hallinge RE Geml J Sánchez-Ramírez S Gonçalves SC Hess J Pringle A (2016) The genus *Amanita* should not be split. Amanitaceae 1: 1–16.

[B30] Tulloss RE Lindgren JE (2005) *Amanitaaprica*–a new toxic species from western North America. Mycotaxon 91: 193–206.

[B31] Tulloss RE Ovrebo CL Halling RE (1992) Studies on *Amanita* (Amanitaceae) from Andean Colombia. Memorials of the New York Botanical Garden 66: 1–46.

[B32] Tulloss RE Yang ZL (2018) (mutable text). Studies in the Amanitaceae http://www.amanitaceae.org [accessed November 2017]

[B33] Vilgalys R Hester M (1990) Rapid genetic identification and mapping of enzymatically amplified ribosomal DNA from several *Cryptococcus* species. Journal of Bacteriology 172: 4238–4246. 10.1128/jb.172.8.4238-4246.1990 2376561 PMC213247

[B34] Wartchow F (2016) *Amanitaviridissima* (Amanitaceae, Basidiomycota), a striking new species from highlands of the semiarid region of Bahia, Brazil. Plant Ecology and Evolution 149: 241–248. 10.5091/plecevo.2016.1198

[B35] Weiß M Yang ZL Oberwinkler F (1998) Molecular phylogenetic studies in the genus *Amanita*. Canadian Journal of Botany 76: 1170–1179. 10.1139/b98-129

[B36] Wolfe BE Tulloss RE Pringle A (2012) The irreversible loss of a decomposition pathway marks the single origin of an ectomycorrhizal symbiosis. PLoS ONE 7: e39597. 10.1371/journal.pone.0039597 PMC3399872 22815710

[B37] Yang ZL (1997) Die *Amanita*-Arten von Südwestchina. Bibliotheca Mycologica 170: 1–240.

[B38] Yang ZL (2004) Two new species of *Amanita* (Basidiomycota) from China. In: Agerer R Piepenbring M Blanz P (Eds) Frontiers in Basidiomycote Mycology.IHW Verlag, Eching, 315–324.

[B39] Yang ZL (2015) Atlas of the Chinese species of Amanitaceae. Science Press, Beijing. [in Chinese]

